# Supplementary material for: Prevalence and patterns of multimorbidity in the Jamaican population: A comparative analysis of latent variable models
Source: PLoS One. 2020 Jul 23;15(7):e0236034. doi: 10.1371/journal.pone.0236034 (PMC7377400; doi:10.1371/journal.pone.0236034)
Supplement: S1 Table — This LCA model excludes obesity. (PDF) [file pone.0236034.s002.pdf]

Null model	vs	Alternative model	*p*-value
1-class		2-class	0.01
2-class		3-class	0.01
3-class		4-class	0.01
4-class		5-class	0.14
