# Supplementary figures and images for: Prevalence and patterns of multimorbidity in the Jamaican population: A comparative analysis of latent variable models
Source: PLoS One. 2020 Jul 23;15(7):e0236034. doi: 10.1371/journal.pone.0236034 (PMC7377400; doi:10.1371/journal.pone.0236034)

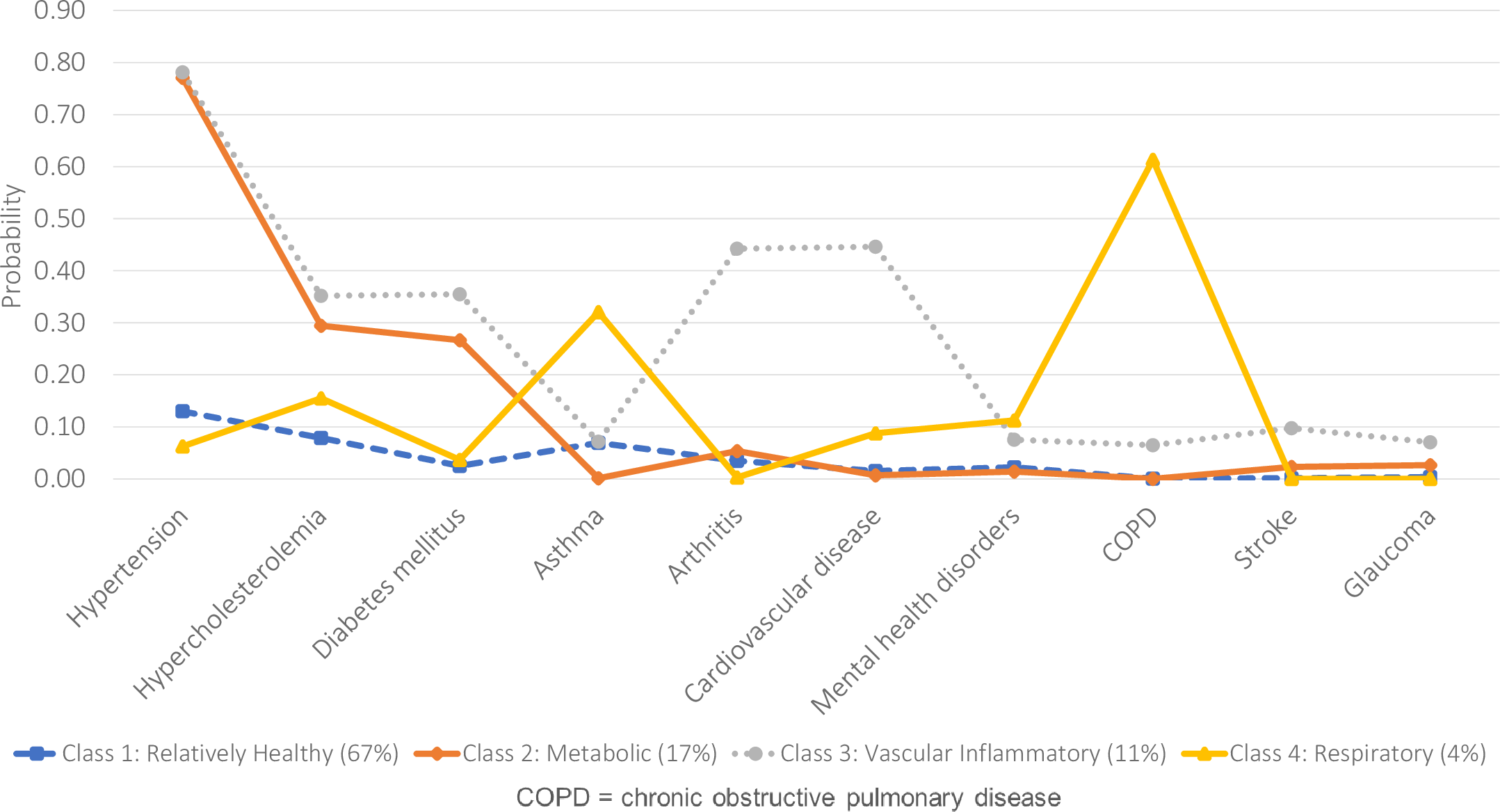

Supplement: S1 Fig — This LCA model excludes obesity. (TIF) [file pone.0236034.s003.tif]
