# Supplementary material for: Prevalence and patterns of multimorbidity in the Jamaican population: A comparative analysis of latent variable models
Source: PLoS One. 2020 Jul 23;15(7):e0236034. doi: 10.1371/journal.pone.0236034 (PMC7377400; doi:10.1371/journal.pone.0236034)
Supplement: Attachment — Submitted filename: Patterns of multimorbidity in the Jamaican population.docx [file pone.0236034.s004.docx]

**Patterns of multimorbidity in the Jamiacan population**

General remark: This article examines the patterns of multimorbidity among the adult population of Jamaica. The authors use advanced statistical techniques to analyze data and the paper is well written. I find the study interesting, but there are some methodological weaknesses in the study that I think should be addressed.

Page 8, line 147 ff.

The following description in the manuscript of the sample weighting procedure could leave the expression that the weighted sample is representative of the Jamaican population:

"The final analytic sample of 2,551 respondents included 790 males and 1,761 females … All analyses were weighted to account for sampling design and non-response as well as differences in the age-sex distribution of the study sample compared to the Jamaican population. Base sampling weights reflected the product of the inverse of the probability of selecting a household and the inverse of the selecting a primary sampling unit, adjusted for non-response. Post-stratification weights were calculated as the number of persons in the Jamaican population between the ages of 15-74 years represented by each individual in the sample within 5-year age-sex categories."

However, when this reviewer calculated the proportion of males using the attached data file, the unweighted percentage of males was 30.97 while the weighted percentage was 31.55 (using the variable sampwt_adj as weight). This indicates that the weighted results are far from representative of the Jamaican adult population (49% of the population were men according to Wilks R, Younger N, Tulloch-reid M, Mcfarlane S, Francis D (2008) Jamaica Health and Lifestyle Survey 2007-8. Kingston: Tropical Medicine Research Institute, University of the West Indies). This could potentially have far-reaching consequences for the quality of the study. The authors should comment on this. Is it possible to generalize the results to the entire population?

Page 8, line 214

"All statistical analyses were carried out via Stata v.15 software, using the LCA Stata Plugin …"

It is well-known that different LCA-programs may come up with different cluster solutions (cf. Haughton, Dominique & Legrand, Pascal & Woolford, Sam. (2009). Review of three latent class cluster analysis packages: Latent gold, poLCA, and MCLUST. The American Statistician. 63. 81-91. 10.1198/tast.2009.0016.)

The most widely used LCA programs are LatentGOLD and MPlus. I tried to repeat the LCA analysis on page 12 using LatentGOLD (without and with weights). The results differ from the results in Table 1, see below:

| File name: | S1 Data.sav | | | | | | | |  |
| --- | --- | --- | --- | --- | --- | --- | --- | --- | --- |
| File size: | 2551 | records | |  |  |  |  |  |  |
| File date: | 2020-feb-6 | 10:06:55 |  |  |  |  |  |  |  |
|  |  | LL | BIC(LL) | AIC(LL) | Npar | L² | df | p-value | Class.Err. |
| UNWEIGHTED  Model1 | 1-Cluster | -2324692 | 4649533 | 4649407 | 11 | 374390,1 | 2036 | 2,9e-78555 | 0 |
| Model2 | 2-Cluster | -2222370 | 4445051 | 4444787 | 23 | 169745,8 | 2024 | 3,4e-34478 | 0,0997 |
| Model3 | 3-Cluster | -2211971 | 4424414 | 4424012 | 35 | 148947,5 | 2012 | 1,7e-30030 | 0,1956 |
| Model4 | 4-Cluster | -2203799 | 4408232 | 4407691 | 47 | 132602,7 | 2000 | 6,3e-26543 | 0,2154 |
| Model5 | 5-Cluster | -2199355 | 4399506 | 4398828 | 59 | 123715 | 1988 | 7,7e-24654 | 0,2047 |
| Model6 | 6-Cluster | -2195780 | 4392518 | 4391702 | 71 | 116565 | 1976 | 1,6e-23137 | 0,2345 |
| Model7 | 7-Cluster | -2193711 | 4388541 | 4387587 | 83 | 112426,7 | 1964 | 5,8e-22265 | 0,3091 |
|  |  |  |  |  |  |  |  |  |  |
| WEIGHTED  Model8 | 1-Cluster | -8247,81 | 16581,92 | 16517,63 | 11 | 1315,148 | 2036 | 1 | 0 |
| Model9 | 2-Cluster | -7876,23 | 15932,89 | 15798,47 | 23 | 571,9874 | 2024 | 1 | 0,1051 |
| Model10 | 3-Cluster | -7836,38 | 15947,31 | 15742,76 | 35 | 492,2788 | 2012 | 1 | 0,1635 |
| Model11 | 4-Cluster | -7810,7 | 15990,08 | 15715,4 | 47 | 440,9214 | 2000 | 1 | 0,221 |
| Model12 | 5-Cluster | -7797,73 | 16058,27 | 15713,46 | 59 | 414,9783 | 1988 | 1 | 0,2792 |
| Model13 | 6-Cluster | -7786,21 | 16129,36 | 15714,41 | 71 | 391,9334 | 1976 | 1 | 0,279 |
| Model14 | 7-Cluster | -7775,15 | 16201,37 | 15716,3 | 83 | 369,8185 | 1964 | 1 | 0,2633 |

In the example with unweighted data, BIC and AIC did not reach a minimum among models with 1-7 latent classes. In the example with the weighted data, a minimum is reached for BIC with a three-cluster solution and for AIC with a five-cluster solution. Compared to this, in Table 1, models with more than four classes are not well identified. I suggest that the authors consider running the analysis again with another LCA program to ensure that results are stable.
